# Symptomatic adjacent-segment epidural hematoma after UNSES for L4/5 lumbar disc herniation: a case report and narrative literature review

**DOI:** 10.3389/fsurg.2026.1961855

**Published:** 2026-09-16

**Authors:** Xueliang Cheng, Rongpeng Dong, Yang Qu

**Affiliations:** Department of Orthopedics, The Second Hospital of Jilin University, Changchun, Jilin, China

**Keywords:** adjacent segment, case report, epidural hematoma, lumbar disc herniation, postoperative complication, spinal endoscopic surgery, UNSES

## Abstract

**Objective:**

To report a rare case of symptomatic adjacent-segment epidural hematoma after uniportal noncoaxial spinal endoscopic surgery (UNSES) for L4/5 lumbar disc herniation and to explore its potential mechanisms.

**Introduction:**

Postoperative symptomatic spinal epidural hematoma (SEH) is an uncommon complication of lumbar surgery and usually occurs at the operative level. Adjacent-segment or remote hematoma after minimally invasive endoscopic lumbar surgery is rare and poorly understood. Continuous intraoperative irrigation and pressure transmission within a confined spinal canal may contribute to this complication.

**Case presentation:**

We reviewed the clinical course, imaging findings, treatment, and outcome of a 56-year-old woman who developed symptomatic adjacent-segment SEH after UNSES for L4/5 lumbar disc herniation. A narrative literature review was also performed to identify similar reports and analyze possible mechanisms.

**Results:**

The patient underwent UNSES decompression and discectomy at L4/5 with marked early symptom relief. One week after operation, she developed recurrent left lower-extremity pain and numbness without progressive motor deficit or sphincter dysfunction. Repeat lumbar magnetic resonance imaging (MRI) showed a small posterior SEH at the adjacent L3/4 level rather than at the index level. Because neurological status remained stable, conservative treatment was continued, resulting in substantial symptom improvement. Literature review suggested that this complication is rare but clinically important. Regarding the mechanism underlying hematoma formation at the adjacent segment, we propose the following hypotheses: transmission of irrigation pressure, pressure disequilibrium between the decompressed segment and the adjacent stenotic segment, and the propagation of occult postoperative bleeding.

**Conclusion:**

Symptomatic adjacent-segment SEH after UNSES is a rare but important postoperative complication. Recurrent radicular symptoms after initial improvement should prompt early MRI and consideration of hematoma at adjacent or remote segments. This case also highlights the need to define a safe irrigation-pressure threshold during minimally invasive spine surgery.

## 1 Introduction

Minimally invasive and endoscopic lumbar decompression techniques have gained widespread acceptance because they reduce soft-tissue injury, blood loss, postoperative pain, and length of hospital stay compared with conventional open surgery (1–6). Among these, unilateral laminotomy with bilateral decompression and unilateral biportal or uniportal endoscopic decompression have become increasingly popular for the treatment of lumbar spinal stenosis and related degenerative disorders (5, 7–9). Although these techniques offer the above advantages, they may also lead to procedure-specific complications with clinical manifestations distinct from those seen in open surgery, such as intracranial hypertension–like syndrome, which has been associated with continuous intraoperative irrigation during minimally invasive procedures (6, 10–13).

Postoperative symptomatic spinal epidural hematoma (SEH) is uncommon but may cause severe neurological deterioration if not recognized and treated promptly (14–16). Most postoperative SEH occur at the operative level and are associated with local surgical bleeding, inadequate hemostasis, coagulation abnormalities, hypertension, or anticoagulant therapy (17–20). In contrast, SEH at an adjacent or remote non-surgical segment is rare. Such cases are diagnostically challenging because recurrent pain or numbness after surgery may be attributed to residual nerve irritation, recurrent disc herniation, postoperative edema, or incomplete decompression.

Here, we report a case of a 56-year-old woman who developed symptomatic adjacent-segment SEH at L3/4 after uniportal noncoaxial spinal endoscopic surgery (UNSES) for L4/5 lumbar disc herniation. The patient had no history of hypertension, anticoagulant use, or coagulation disorder and improved after conservative treatment. We also review the relevant literature and discuss possible mechanisms, diagnostic strategies, and treatment considerations.

## 2 Case presentation

A 56-year-old woman presented with low back pain and left lower-extremity radicular pain of more than 2 months’ duration, with worsening during the week before admission. She had no history of hypertension, coagulopathy, or anticoagulant/antiplatelet use. The patient underwent three months of conservative treatment, with no significant symptom relief. Examination showed hypoesthesia over the lateral left lower leg and dorsum of the foot, grade 4/5 strength of the left extensor hallucis longus, a diminished left Achilles reflex, and a positive straight leg raising test on the left. No bowel or bladder dysfunction was present. Preoperative images showed loss of normal lumbar lordosis, and a left paracentral L4/5 disc herniation compressing the dural sac and corresponding nerve root, consistent with left-sided radiculopathy (Figure 1a). The patient underwent UNSES decompression and discectomy at L4/5. Operative time was 40 min, estimated blood loss was less than 50 mL, and no dural tear, vascular injury, or abnormal bleeding was observed intraoperatively. The drainage tube was removed 24 h postoperatively because the patient had minimal drainage output. Early after surgery, the patient reported marked relief of low back and radicular pain, without new neurologic deficit. One week after operation, however, she developed recurrent left lower-extremity pain and numbness without a clear precipitating event. Muscle strength of left ankle dorsiflexion was graded as IV, while strength in the other muscle groups was normal. No bladder or bowel dysfunction was noted. Considering the possibility of postoperative symptom rebound, the patient underwent treatment with intravenous dexamethasone (10 mg) and mannitol (50 g), along with oral administration of mecobalamin and other medications. After a week of treatment, no significant improvement in symptoms was observed. Repeat lumbar Magnetic Resonance Imaging (MRI) demonstrated MRI revealed a spindle-shaped space-occupying lesion in the posterior epidural space, extending from the inferior border of L3 to of L4 over an approximate craniocaudal length of 3 cm (Figure 1b). The lesion showed isointense on T1-weighted images and hyperintense on T2-weighted images. It produced significant compression of the dural sac. Considering the lesion's epidural location, acute onset of neurological symptoms, MRI appearance, the SEH was considered the most likely diagnosis. Further review disclosed no coagulopathy, perioperative anticoagulant exposure, or other identifiable precipitating factor. Because the hematoma was small, no progressive neurologic deficit or sphincter dysfunction was present and the patient declined reoperation, conservative management was continued. The patient was informed that close clinical monitoring would be required and that surgical treatment would be considered if neurological function or imaging findings worsened. After an additional 1 weeks of follow-up, repeat MRI showed no significant enlargement of the hematoma compared with 1 week earlier (Figure 1c). The patient's symptoms had improved. Muscle strength had nearly returned to normal, and no apparent limitation in mobility was observed. At the 28-day postoperative follow-up, during the telephone follow-up, we confirmed that the patient's clinical symptoms had improved significantly, with almost no pain and a VAS score of 1. However, the patient was unwilling to return to the hospital for further follow-up and relevant imaging examinations.

**Figure 1:**
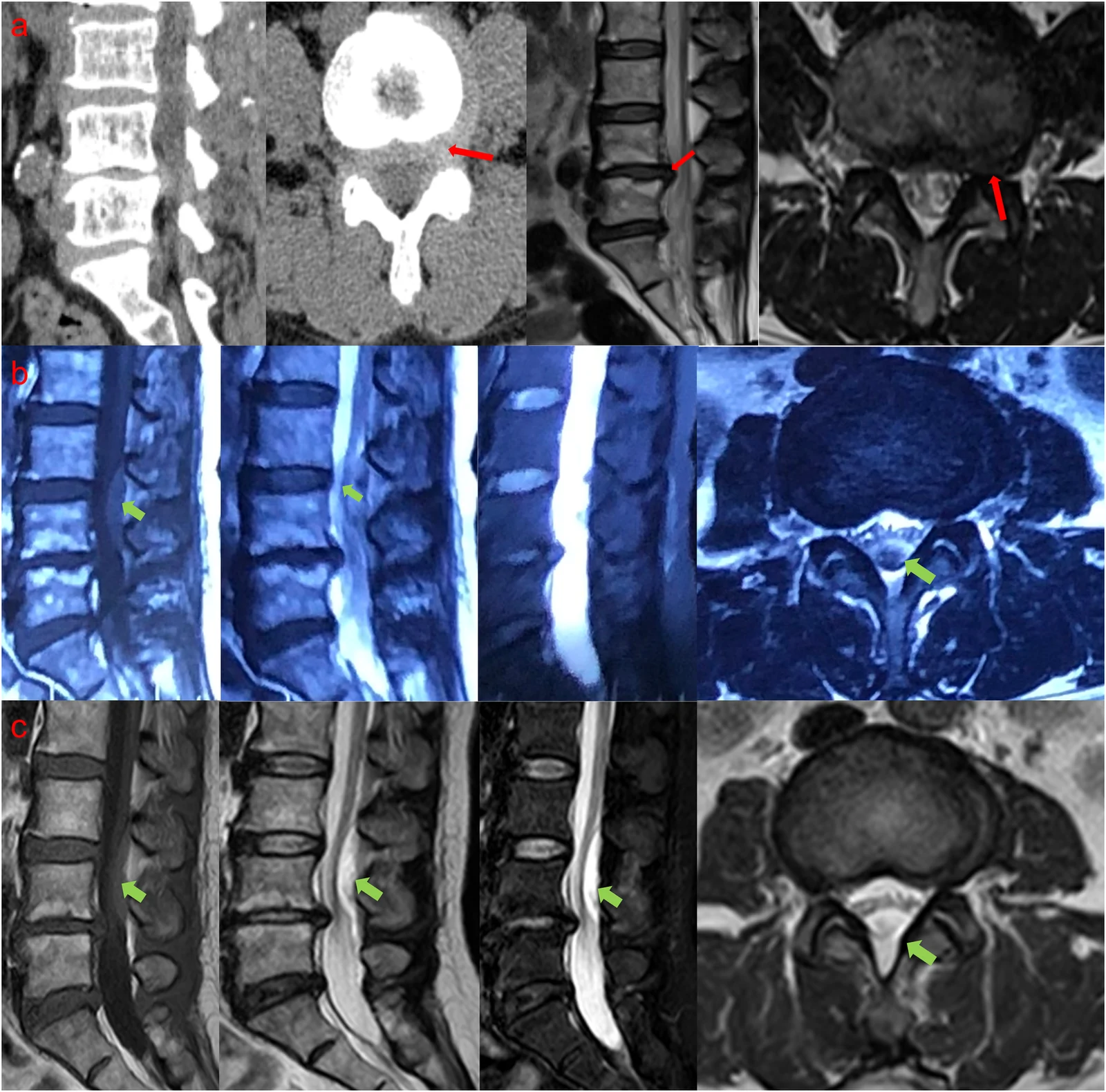
**(a)** preoperative images show a left-sided L4/5 disc herniation compressing the dural sac and corresponding nerve root; **(b)** lumbar MRI obtained 2 weeks postoperatively show a long oval-shaped hematoma in the posterior epidural space of the spinal canal behind the L3-L4 vertebral bodies. **(c)** Lumbar MRI obtained 3 weeks postoperatively show no significant enlargement of the hematoma compared with 1 week earlier. Red arrow: the herniated disc responsible for the neurological symptoms; Green arrow: epidural hematoma.

## 3 Timeline

A table showcasing a timeline with relevant data from the episode of care (Table 1).

**Table 1:** Timeline of the episode of care.

Time point	Clinical event and relevant findings	Diagnostic assessment	Treatment/intervention	Outcome
Preoperative period	Patient presented with symptoms caused by lumbar degenerative disease/disc herniation.	Clinical examination and lumbar MRI confirmed the surgical indication.	UNSES was planned.	Patient was considered suitable for endoscopic surgery.
Day 0	UNSES was performed at the affected lumbar level.	No immediate intraoperative complication was reported.	Endoscopic decompression/discectomy was completed.	Initial postoperative recovery was satisfactory.
Day 7	Recurrent left lower-extremity pain and numbness	Postoperative symptom rebound caused by prolonged neural compression.	Conservative treatment, including anti-inflammatory therapy, dehydration therapy, and neurotrophic medication	No meaningful improvement
Day 14	The above symptoms showed no significant improvement.	Postoperative MRI was obtained and adjacent-segment intraspinal hematoma was identified.	Conservative treatment was selected according to the patient's clinical status.	Neurological status and hematoma progression were monitored.
Day 21	The symptoms had improved.	Repeat MRI was obtained	The patient continued to receive conservative treatment.	MRI showed no significant enlargement of the hematoma
Day 28	The patient was reassessed clinically and radiologically.	Follow-up imaging evaluated hematoma resolution and spinal decompression.	Continued observation and rehabilitation as appropriate.	Neurological function and symptoms improved, with no further deterioration reported.

## 4 Discussion

Postoperative symptomatic intraspinal hematoma is a rare but potentially serious complication of lumbar decompression surgery. In most cases, clinical attention is directed toward hematoma at the index level, where direct tissue manipulation and epidural venous injury are expected. However, the present case highlights a less recognized entity: adjacent-segment or remote intraspinal hematoma after minimally invasive spinal surgery. This pattern is clinically important because symptom recurrence after initial postoperative improvement may be misattributed to insufficient decompression, recurrent disc herniation, or routine postoperative irritation, thereby delaying diagnosis.

The hematomas reported in the literature following spinal minimally invasive surgery at non-operated levels include SEH and spinal subdural hematomas (SSDH). SEH are typically located between the dural sac and the osseous structures of the spinal canal and may originate from bleeding of the epidural venous plexus, paravertebral soft tissues, or adjacent bony structures. SSDH, in contrast, are located between the dura mater and the arachnoid mater; their development may be associated with dural injury, changes in subarachnoid pressure caused by cerebrospinal fluid leakage, or rupture of bridging veins or small blood vessels. Although these two types of hematoma differ in anatomical location, imaging characteristics, and clinical management, both may exert mass effects on the spinal cord, cauda equina, or nerve roots, resulting in worsening postoperative pain, sensory disturbances, and muscle weakness, and, in severe cases, bladder or bowel dysfunction. Collectively, these reports suggest that postoperative intraspinal hematoma after minimally invasive lumbar surgery is uncommon but not invariably confined to the operative level, and may involve adjacent or remote compartments (Tables 1, 2).

**Table 2:** Summary of reported intraspinal hematoma after minimally invasive spine surgery.

Patient	Surgical procedure	Hematoma type	Location	Time of onset	Main clinical manifestations	Management	Outcome	Ref.
82-year-old female	UBE /L4-5	SEH	T12-L4	Postoperative day 3	Minimal lower limb pain	Observation	Full recovery	(14)
62-year-old female	UBE /L4-5	SEH	L2-5	Postoperative day 3	Severe lower back pain, radiating pain	Surgery	Full recovery	(21)
71-year-old male	PELD /L2-3	SEH	L1-S1	Immediately postoperatively	Severe radiating pain	Surgery	Partial recovery	(22)
76-year-old female	UBE /L3-4	SSDH	L1-2	Immediately postoperatively	Left leg monoplegia	Intravenous steroids	Full recovery	(23)
88-year-old male	UBE /L4-5	SSDH	L2-4	Postoperative day 1	None	Observation	Full recovery	(23)
68-year-old female	IELD /L4-5	SSDH	L5/S1	Postoperative day 2	Radiating pain urinary retention	Surgery	Full recovery	(24)
76-year-old male	MD / L3-5	SSDH	T10-L3	Postoperative day 2	Weakness,sensory changes, urinary retention	Surgery	Full recovery	(25)
80-year-old male	MD / L4-5	SSDH	L1-S1	Immediately postoperatively	Lower extremity numbness	Conservative treatment	Full recovery	(26)

UBE, unilateral biportal endoscopy; SEH, spinal epidural hematoma; SSDH, spinal subdural hematomas; PELD, percutaneous endoscopic transforaminal lumbar discectomy; IELD, inter-laminar endoscopic lumbar decompression; MD, microdiscectomy.

The precise mechanism underlying this complication remains uncertain; however, continuous intraoperative irrigation may be an important precipitating factor1 (13, 15, 24). Unlike conventional microdiscectomy, currently used minimally invasive spinal endoscopic techniques, including uniportal coaxial endoscopy, unilateral biportal endoscopy (UBE), and unilateral non-coaxial endoscopic techniques, all rely on continuous saline irrigation to maintain a clear operative field, remove surgical debris, and control minor bleeding. Although irrigation offers substantial technical advantages, this fluid environment may also generate non-physiological fluctuations in intraspinal pressure, particularly in patients with severe spinal canal stenosis or limited epidural compliance (Figure 2a). In the confined space of a stenotic spinal canal, irrigation pressure may not dissipate uniformly and instead may be transmitted cranially or caudally through the epidural space, with accumulation at adjacent or even remote levels where egress is limited. Based on these considerations, we propose a hypothesis regarding the mechanism of adjacent-segment hematoma formation: transient pressure overload may cause rupture of the epidural venous plexus, thereby triggering hematoma development. Because these veins are thin-walled and valveless, they are particularly susceptible to changes in hydrostatic and venous pressure. During endoscopic surgery, continuous irrigation may temporarily compress epidural veins and obscure overt bleeding. However, when outflow is intermittently impeded, the working channel is crowded by instruments, pump pressure fluctuates, or the stenotic canal behaves as a semi-enclosed compartment, local pressure may increase abruptly or vary substantially, predisposing fragile venous structures to rupture. This mechanism may be especially relevant at adjacent segments, where tissue compliance is lower and local pressure gradients may be greatest. Pressure transmitted along tissue planes may therefore result in either frank venous rupture or occult venous oozing within the spinal canal (Figure 2b).

**Figure 2:**
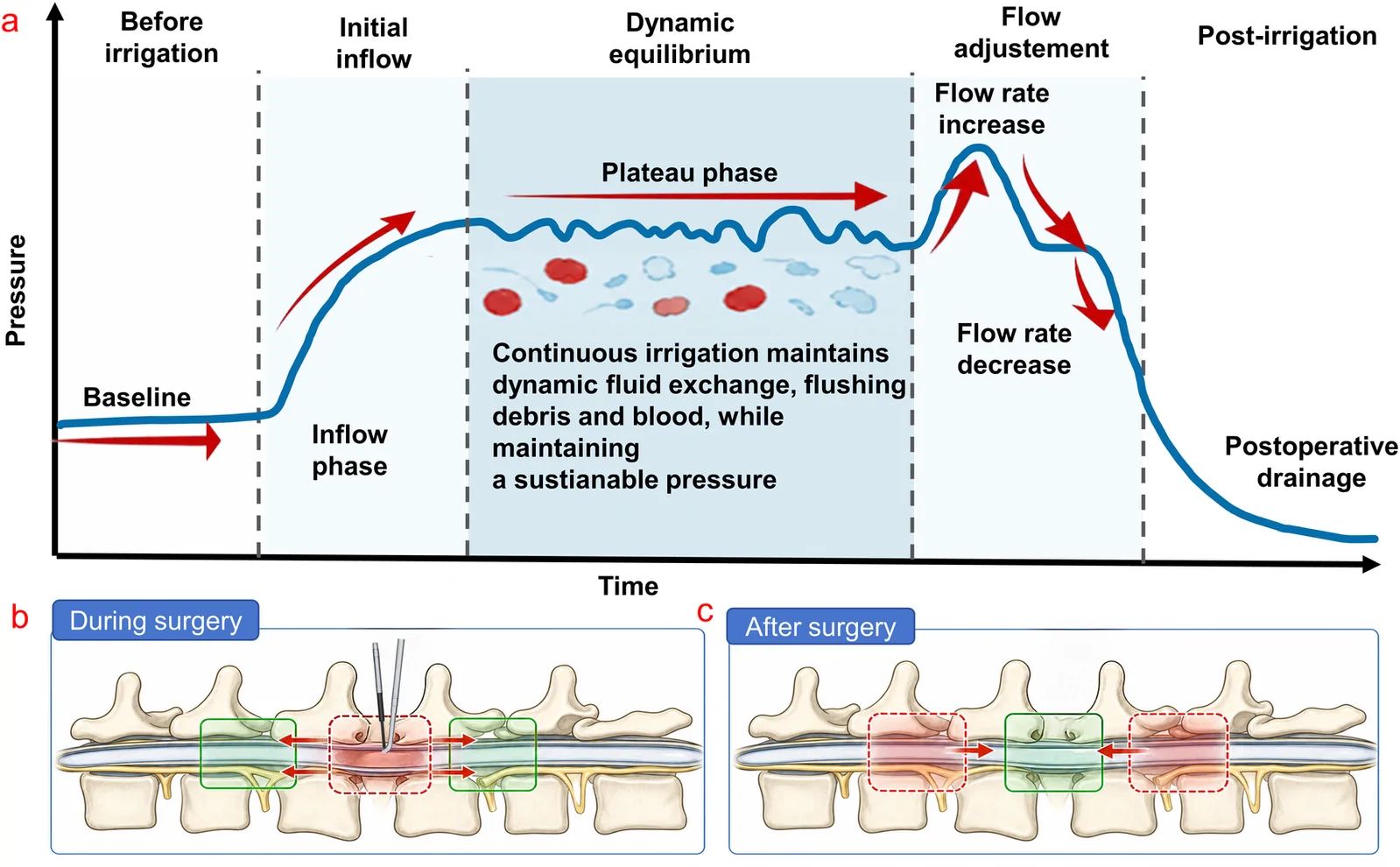
Conceptual schematic illustrating the proposed, hypothetical relationship between irrigation-related pressure transmission and the potential risk of postoperative epidural hematoma. This schematic is not based on measured intraoperative pressure data. **(a)** Changes in intraspinal irrigation pressure in the surgical area before, during, and after surgery. **(b)** Intraoperative irrigation pressure in the surgical area and adjacent segment. **(c)** Irrigation pressure in the surgical area and adjacent segment after surgery. Red dashed box: the high-pressure zone; Green solid box: the low-pressure zone, Red arrow: the direction of pressure transmission.

The second hypothesized mechanism is that adjacent-segment hematoma formation may be associated with the pressure-compartment effect in the degenerative stenotic lumbar canal (22). Prior studies have suggested that irrigation can alter epidural pressure, while decompression itself further disrupts the preexisting pressure balance within a relatively closed space. In patients undergoing minimally invasive decompression, the index level is often severely stenotic, and adjacent segments frequently demonstrate varying degrees of concomitant narrowing. Under continuous irrigation, the decompressed level may become relatively more compliant, whereas adjacent stenotic levels remain constrained, creating a pressure gradient at the transition zone between these regions (Figure 2C). This gradient may be further amplified by postoperative closed-suction drainage (Figure 2A). In theory, such pressure disequilibrium may increase venous wall stress, promote venous disruption, and lead to hematoma formation within a local potential space. Certainly, both proposed mechanisms currently lack direct supporting data. The relationship between irrigation-related pressure transmission and adjacent-segment hematoma formation remains hypothetical and should be confirmed by future studies incorporating direct intraoperative pressure monitoring, as well as additional clinical and experimental evidences.

A third possible mechanism is the gradual accumulation of occult postoperative bleeding. Minor oozing from bone, small epidural veins, or soft tissue at the operative site may spread longitudinally through potential intraspinal spaces and collect at an adjacent level, resulting in hematoma formation. Previous case reports have described “spreading-type” epidural hematomas and postoperative hematomas extending across multiple segments, indicating that blood may propagate along the epidural space rather than remain restricted to the surgical plane (15, 27). In our patient, follow-up MRI demonstrated an abnormal posterior signal extending from the inferior margin of L3 to the inferior margin of L4, suggesting that blood may have originated from the L4/5 operative level and then extended cranially through the laminotomy site, the epidural fat plane, or the corridor created after ligamentum flavum resection, eventually accumulating at the adjacent L3/4 level to form a small hematoma.

Reported cases suggest that hemorrhagic involvement is not necessarily confined to the operative level. These hematomas most often occur in the early postoperative period, typically within 1–3 days, but delayed presentation after transient symptom relief has also been described. In some patients, symptoms recur only after drain removal, ambulation, or even discharge, indicating a potentially occult and delayed course. Clinical severity varies widely, ranging from worsened pain or numbness to motor weakness, gait disturbance, and bladder dysfunction, although some patients remain neurologically intact despite multilevel hematoma on imaging. Management should be guided primarily by neurological status and the degree of radiographic compression. Urgent surgical evacuation is generally required in patients with progressive neurological deficits or cauda equina-related symptoms, whereas conservative treatment may be appropriate in carefully selected neurologically stable patients. Overall, favorable outcomes have been reported with both surgical and nonoperative management when diagnosis is timely and treatment is individualized.

This case also has preventive implications. Although definitive risk factors cannot be established from isolated reports, several technical considerations appear reasonable: avoiding unnecessarily high irrigation pressure, ensuring adequate fluid egress throughout the procedure, minimizing prolonged pressure buildup in a confined canal, and achieving meticulous hemostasis before closure. In patients with severe stenosis, multilevel degenerative narrowing, adhesions, or vascular fragility, prompt lumbar MRI is warranted if symptoms recur.

Several limitations must be acknowledged. First, the current evidence base consists almost entirely of case reports and small case series, precluding reliable estimates of incidence or causality. Second, direct intraoperative proof of the bleeding source is usually lacking, so the proposed pressure-related mechanism remains inferential. Third, in the present study, postoperative follow-up focused mainly on the patient's subjective symptoms and perceptions. This approach may have limited our ability to comprehensively assess neurological recovery and functional outcomes. Future evaluations should combine subjective pain measures with validated patient-reported outcome instruments and objective assessments, including pressure-pain sensitivity, muscle strength, sensory function, and gait ability, to provide a more comprehensive assessment of postoperative recovery (28). Forth, the literature includes both SEH and SSDH, which are anatomically distinct entities, although they may share overlapping pressure-mediated pathophysiologic pathways in the setting of minimally invasive fluid-based surgery. Nonetheless, the repeated observation of adjacent-level or remote hematoma after endoscopic procedures, often without overt dural injury, supports the biological plausibility of this hypothesis.

## 5 Conclusion

Adjacent-segment or remote intraspinal hematoma after minimally invasive lumbar surgery appears to be rare but clinically meaningful. Their underlying mechanisms remain unclear. We therefore propose several potential possible mechanisms, including transmission of irrigation pressure, pressure disequilibrium between the decompressed segment and the adjacent stenotic segment, and the gradual accumulation of occult postoperative bleeding. Recurrent postoperative radicular symptoms after initial improvement should prompt consideration of not only recurrent disc pathology or inadequate decompression at the index level, but also hematoma at adjacent or remote segments. Early MRI is essential for diagnosis, and treatment should be determined according to neurological progression and the degree of neural compression. Meanwhile, this case highlights an important issue for future research: the pressure distribution within the operative field and adjacent spinal segments during minimally invasive spine surgery, as well as the safe threshold for intraoperative irrigation pressure. Further investigation is needed to address these questions.

## 6 Patient perspective

I had persistent symptoms that did not improve after three months of conservative treatment. Because the symptoms continued to affect my daily life, I decided to undergo surgery. After surgery, I experienced significant pain and distress when a hematoma developed again. The recurrence was unexpected and made the postoperative recovery especially difficult. I was grateful that the rare hematoma was recognized promptly through careful clinical evaluation and imaging, allowing timely treatment before more serious complications occurred. This experience highlighted the importance of close postoperative monitoring and rapid assessment of new or worsening symptoms.

## Data Availability

The original contributions presented in the study are included in the article/Supplementary Material, further inquiries can be directed to the corresponding author.
